# Characteristics of attitude and recommendation of oncologists toward exercise in South Korea: a cross sectional survey study

**DOI:** 10.1186/s12885-015-1250-9

**Published:** 2015-04-10

**Authors:** Ji-Hye Park, Minsuk Oh, Yong Jin Yoon, Chul Won Lee, Lee W Jones, Seung Il Kim, Nam Kyu Kim, Justin Y Jeon

**Affiliations:** 1 Department of Sport and Leisure Studies, Yonsei University, 50 Yonsei-Ro, Seodaemun-Gu, Seoul 120-749 Korea; 2 Memorial Sloan Kettering Cancer Center, New York, NY USA; 3 Division of Breast Cancer Surgery, Department of Surgery, Breast Cancer Clinic, Severance Hospital, Yonsei University College of Medicine, 50 Yonsei-ro, Seodaemun-gu, Seoul 120-752 Korea; 4 Division of Colorectal Surgery, Department of Surgery, Colorectal Cancer Clinic, Severance Hospital, Yonsei University College of Medicine, 50 Yonsei-ro, Seodaemun-gu, Seoul 120-752 Korea

**Keywords:** Physical activity recommendations, Oncologist, Cancer

## Abstract

**Background:**

The purpose of the present study was to examine 1) characteristics and attitudes of oncologists toward exercise and toward recommending exercise to their patients, 2) association among oncologists’ own physical activity levels, exercise recommendations, and their attitudes toward recommending exercise.

**Methods:**

A total of 167 oncologists participated in this survey study (41 surgeons, 78 medical oncologists, 25 radiation oncologists, and 21 others). Most oncologists included in the study treat more than one type of cancer, including colorectal, gastric, breast, lung, and liver cancer. To analyze the data, the one-way ANOVA, and *t*-test were used. All data were indicated for mean, SD, and proportions.

**Results:**

Most oncologists agreed that exercise is beneficial (72.8%) and important (69.6%), but only 39.2% of them agreed that exercise is safe, and only 7.2% believed that cancer patients manage to exercise during cancer treatment. Forty-six percentage of the surveyed oncologists recommended exercise to their patients during the past month. The average amount of participation in physical activity by oncologists who participated in the study was 139.5 ± 120.3 min per week, and 11.4% of the study participants met the American College of Sports Medicine (ACSM) guidelines. Oncologists’ own physical activity levels were associated with their attitudes toward recommending exercise. Belief in the benefits of exercise in the performance of daily tasks, improvement of mental health, and the attenuation of physical decline from treatment were the three most prevalent reasons why oncologists recommend exercise to their patients. Barriers to recommending exercise to patients included lack of time, unclear exercise recommendations, and the safety of patients.

**Conclusions:**

Oncologists have favorable attitudes toward exercise and toward recommending exercise to their patients during treatment. However, they also experience barriers to recommending exercise, including lack of time, unclear exercise guidelines for cancer patients, and concerns regarding the safety of exercise.

## Background

Cancer has been the number one cause of death since 1983 and poses a major public health concern in Korea [1]. The incidence rate of all cancers showed an annual increase of 3.3% between 1999 and 2009 [1]. There were 192,561 cancer cases and 69,780 cancer deaths in 2009 in Korea [1]. The cumulative risk of developing cancer during a lifetime was 37.9% for men and 32.7% for women [1]. Despite notable improvement in five-year relative survival rates for all cancers in Korea [2], a large number of cancer patients still experience cancer recurrence. Therefore, identifying the factors that contribute to cancer recurrence and survival is important.

Beneficial effects of exercise and physical activity on health-related fitness, quality of life, and other patient-reported outcomes among cancer survivors during and after treatment have been reported previously [3-11]. A recent meta-analysis reported that physical activity is associated with reduced all-cause, breast cancer–specific, and colon cancer–specific mortality [12,13]. In addition to the benefits of physical activity on the health of cancer survivors, the American College of Sports Medicine (ACSM) reported that participation in exercise and physical activity is safe during and after adjuvant cancer therapy [14].

Despite these and other well documented benefits of exercise and physical activity and the safety of participating in exercise, many cancer patients still remain physically inactive [15]. Only 29.6% of cancer survivors met the exercise recommendations of the ASCM [16], significantly less than the percentage among the non-cancer population. Another meta-analysis also reported that 53–72% of cancer survivors do not meet ACSM exercise guidelines for cancer patients [17]. Although we have recently identified that there were no significant difference in total physical activity time between pre-diagnosis and on-treatment among colorectal cancer patient [18], participation in exercise declines substantially during cancer treatment and may not return to pre-diagnosis levels of exercise after treatment is completed [15]. Therefore, it is important to find strategies to increase exercise participation in cancer patients.

Jones et al. [17] reported that simple oncologist’s physical activity recommendation increased their patient’s physical activity levels in women newly diagnosed with breast cancer. Another study found that a physician’s recommendation regarding physical activity has been demonstrated to be a strong predictor of a patient’s level of physical activity [19,20]. Nevertheless, although cancer patients are highly motivated to receive exercise information from their oncologists [19,20] and oncologists have a favorable view on exercise for their patients, most oncologists still do not recommend exercise for their patients [16,17,21,22]. However, barriers to oncologists’ recommendation of exercise to their patients have not been studied fully. Furthermore, the oncologists’ attitude toward recommending exercise and practice of exercise recommendations to their cancer patients has not been studied. Furthermore, whether factors such as their attitudes toward exercise and their own physical activity levels may influence oncologists’ recommendations toward exercise and the barriers they experience in recommending exercise have not been studied.

The purposes of this study were to evaluate 1) the attitudes of oncologists toward recommending exercise, 2) the association between oncologists’ own physical activity levels and the attitudes of oncologists toward recommending exercise, and 3) the barriers experienced by oncologists in recommending exercise to their patients.

## Methods

### Ethical considerations

The study was approved by the Ethics Review Committee of Severance Hospital.

### Participants and procedure

The goal of the study was to approach all oncologists in South Korea. Our first strategy was to distribute questionnaires at the annual conference of Korean Cancer Association (18th November 2011, Lotte Hotel, Seoul, Korea). A total of 202 questionnaires were distributed and 44 questionnaires were returned (21.7%). Our second strategy was to collect e-mail addresses from webpage of hospitals. Through this effort, we have collected 386 e-mails addresses of oncologists in South Korea. We sent questionnaires to 386 oncologists via e-mails and a total of 123 questionnaires were successfully filled out on-line (28.9%).

### Study instruments

The attitudes of oncologists toward exercise and toward recommending exercise for cancer patients were assessed by a questionnaire developed and tested by Jones et al. [21]. Except two items (intention, behavior), all items in the questionnaire were scored in 1 to 7 point Likert scales (agree to disagree). To analyze the survey question, all items were grouped into three categories: disagree (1 to 2 point), neutral (3 to 5 point), agree (6 to 7 point), and the proportion category was used for the intention and behavior item (0 to 33, 33 to 67, 67 to 100%) [23,24]. The questionnaire is explained in detail in a previous publication [21]. To measure oncologists’ perceptions of the benefits of exercise and of barriers to recommending exercise to patients, a questionnaire developed by Karvinen et al. was utilized [25]. These questionnaires were translated into Korean and then back-translated into English by a bilingual university professor (J.Y.J.). Then, three university professors (J.Y.J., L.J.W., C.S.H.) who understood the nature of the study and were fluent in both Korean and English held an expert reviewers’ meeting to establish translation validity. To measure the level of physical activity of oncologists, the Godin Leisure -Time Exercise Questionnaire (GLTEQ; Strenuous exercise: running, hockey, football, soccer, squash, basketball, cross, judo, roller skating, vigorous swimming, Moderate exercise: fast walking, baseball, tennis, easy bicycling, volleyball, badminton, easy swimming, alpine skiing, popular and folk dancing, Mild exercise: yoga, archery, fishing from river bank, bowling, horseshoes, golf, snow-mobiling, easy walking) [26,27] was used.

### Statistical analyses

All analyses were conducted using SPSS version 18.0. All data that normality of distribution in this study is presented as mean ± standard deviation (SD). The normality of distribution was verified with Shapiro-Wilk test. Oncologists’ characteristics and attitudes toward exercise and toward recommending exercise during cancer treatment were analyzed using descriptive statistics. To analyze the relationship between oncologists’ own physical activity levels and their attitudes toward exercise and toward recommending exercise, we have divided our participants into three groups using the 33.3% and 66.6% cut-offs to generate tertiles based on each individual’s physical activity level. Differences in attitude toward exercise and attitude toward recommending exercise among three groups were analyzed by one-way ANOVA. To test the difference in percentage of exercise recommendation based on oncologists’ perceived benefits of exercise and barriers for cancer survivors were compared by using a *t*-test. The significance level of the difference was set as p < 0.05.

## Results

The demographic characteristics of the participants are summarized in Table 1. In brief, the mean age of respondents was 42.99 ± 8.55 years; 66.5% were male; 24.6, 46.7, 15.0, 12.6% were surgeons, medical oncologists, radiation oncologists, and other respectively; and they had a mean of 10.51 ± 7.95 years in practice. Among oncologists who participated in the study, 11.4% met the current ACSM exercise guidelines for healthy adults: under age 65 with no apparent chronic disease or condition (i.e., at least 150 min of moderate intensity physical activity per week).

**Table 1:** **Demographic characteristics**

Variable		No. of respondents	%	Mean ± SD
Age (years)		167		43.0 ± 8.6
Number of years in practice		167		10.5 ± 8.0
Sex	Male	111	66.5	-
	Female	56	33.5	
Specialty	Surgeon	41	24.6	-
	Medical oncology	78	46.7	
	Radiation oncology	25	15.0	
	Other	21	12.6	
Type of cancer patients (Multiple response) No:275	Colorectal cancer	63	22.9	-
	Gastric cancer	51	18.5	
	Breast cancer	47	17.0	
	Lung cancer	46	16.7	
	Liver cancer	27	9.8	
	Other	41	14.9	
How many cancers do you take care of	One cancer	91	54.5	-
	Two cancers	47	28.1	
	Over 3 cancers	25	15.1	
Exercise (min/week)	Mild exercise	167		80.1 ± 85.6
	Moderate exercise	167		44.1 ± 62.8
	Vigorous exercise	167		15.4 ± 48.1
	Total exercise	167		139.5 ± 120.3
	Meeting exercise guidelines	19	11.4%	

Values given as mean ± SD for continuous variables and frequency (%) for categorical variables.

ACSM guidelines: At least 150 minutes of vigorous to moderate intensity physical activity per week.

### Attitudes toward exercise and toward recommending exercise for cancer patients

Overall, a majority of the surveyed oncologists agreed that exercise is beneficial (72.8%) and important (69.6%) for patients with cancer during treatment. But only 39.2% of the oncologists agreed that exercise for cancer patients is safe. Fifty-five percentage of oncologists agreed that their patients believe that they should exercise during treatment. However, only 31.2% of the oncologists believed that their fellow oncologists think that their patients should exercise during treatment. Similarly, 39.3% of oncologists agreed that patients are capable of exercise during treatment, although only 12% agreed that it would be easy for patients to exercise at this time.

Furthermore, 40.7% of oncologists agreed that providing an exercise recommendation would be well received by their patients, while only 29.3% agreed that their patients would follow this recommendation. Similarly, 59.9% and 40.7% of oncologists agreed that their fellow oncologists and patients, respectively, thought that they should provide an exercise recommendation during cancer treatment. Thirty percentage of oncologists agreed that providing an exercise recommendation was within their control, and 31.7% agreed that it would be easy to provide an exercise recommendation to their patients during treatment. Fifty percentage of oncologists agreed that they tried to recommend exercise to their patients when appropriate. Lastly, 37.6% of oncologists believed that less than 33% of their patients attempt to exercise during treatment, while 60% believed that less than 33% of their patients actually manage to exercise during treatment (Table 2).

**Table 2:** **Attitudes toward exercise and recommending exercise for cancer patients**

Survey item		Mean ± SD	Disagree		Neutral		Agree	
			N	%	N	%	N	%
Attitudes toward exercise	In my opinion exercise is beneficial during treatment.	5.9 ± 1.4	5	4.0	28	22.4	91	72.8
	In my opinion exercise is important during treatment.	5.9 ± 1.3	5	4.0	32	25.6	87	69.6
	In my opinion exercise is safe during treatment.	5.1 ± 1.3	3	2.4	71	56.3	49	39.2
	Most patients believe they should exercise during cancer treatment.	5.38 ± 1.41	5	4.0	51	40.8	69	55.2
	Most fellow oncologists think patients should exercise during cancer treatment.	4.8 ± 1.4	10	8.0	76	60.8	39	31.2
	Most of my patients are capable of exercising during cancer treatment.	4.9 ± 1.5	9	7.2	67	53.6	49	39.3
	Exercising during treatment for my patients is easy.	3.9 ± 1.1.4	10	15.2	91	72.8	15	12.0
Attitudes toward recommending exercise	Providing an exercise recommendation would be well received.	5.1 ± 1.2	3	1.8	96	57.5	68	40.7
	If I provided a recommendation, patients would follow my advice.	4.9 ± 1.1	3	1.8	115	68.9	49	29.3
	My fellow oncologists think I should recommend exercise.	5.6 ± 1.3	6	3.6	61	36.5	100	59.9
	My patients think I should recommend exercise.	5.1 ± 1.4	8	4.8	91	54.5	68	40.7
	Whether I recommend exercise is completely up to me.	4.6 ± 1.5	17	10.2	99	59.3	50	29.9
	When appropriate, I try to recommend exercise.	4.6 ± 1.6	21	12.6	93	55.7	53	31.7
	For me, providing a recommendation is easy.	5.1 ± 1.4	9	5.4	83	49.7	75	44.9
			0 ~ 33%		34 ~ 67%		68 ~ 100%	
	What % of your patients in your opinion try to exercise during cancer treatment?	44.8 ± 20.5	47	37.6	51	40.8	19	15.2
	What % of your patients in your opinion manage to exercise during cancer treatment?	33.6 ± 19.0	75	60	31	24.8	9	7.2

Values given as mean ± SD for continuous variables and frequency (%) for categorical variables. All items rated on 7-point Likert scale: Disagree (responses 1–2), Neutral l (responses 3–5), Agree (responses 6–7).

### Attitudes toward exercise and toward recommending exercise across oncologists’ own physical activity levels

To assess the association between oncologists’ own physical activity participation levels and their attitudes toward exercise and toward recommending exercise, we divided the oncologists into three groups according to their own physical activity levels (Tertiles). The analyses showed that oncologists’ own physical activity levels were not associated with their attitudes toward exercise but were associated with their attitudes toward recommending exercise. Compared with oncologists who participated in very minimal amounts of physical activity, those who were more physically active believe that their fellow oncologists think that they should recommend exercise to their patients. In addition, more physically active oncologists also think that their patients believe that they should recommend exercise (Table 3).

**Table 3:** **Attitudes toward exercise and toward recommending exercise across oncologists’ own physical activity levels**

		Physical activity level (Tertiles)		
		Low (N = 56)	Middle (N = 52)	High (N = 58)
Attitudes toward exercise	In my opinion exercise is beneficial during treatment.	5.80 ± 1.43	5.85 ± 1.35	5.93 ± 1.39
	In my opinion exercise is important during treatment.	5.64 ± 1.50	5.90 ± 1.11	6.02 ± 1.20
	In my opinion exercise is safe during treatment.	5.00 ± 1.34	5.22 ± 1.27	5.21 ± 1.25
	Most patients believe they should exercise during cancer treatment.	5.21 ± 1.46	5.25 ± 1.49	5.67 ± 1.25
	Most fellow oncologists think patients should exercise during cancer treatment.	4.68 ± 1.39	4..70 ± 1.49	5.02 ± 1.32
	Most of my patients are capable of exercising during cancer treatment.	4.71 ± 1.59	4.81 ± 1.36	5.17 ± 1.47
	Exercising during treatment for my patients is easy.	3.71 ± 1.40	4.04 ± 1.39	3.88 ± 1.42
Attitudes toward recommending exercise	Providing an exercise recommendation would be well received.	5.14 ± 1.17	5.04 ± 1.14	5.19 ± 1.15
	If I provided a recommendation, patients would follow my advice.	4.79 ± 1.14	4.83 ± 1.11	4.93 ± 1.11
	My fellow oncologists think I should recommend exercise.	5.43 ± 1.26	5.36 ± 1.43	5.91 ± 1.22
	My patients think I should recommend exercise.	4.73 ± 1.40	5.04 ± 1.45	5.40 ± 1.38*
	Whether I recommend exercise is completely up to me.	4.18 ± 1.54	4.48 ± 1.42	4.98 ± 1.32*
	When appropriate, I try to recommend exercise.	4.50 ± 1.50	4.57 ± 1.54	4.78 ± 1.64
	For me, providing a recommendation is easy.	4.80 ± 1.37	5.04 ± 1.44	5.55 ± 1.33*
Exercise recommendations	Exercise recommendation to their cancer patients.	41.80 ± 24.63	41.73 ± 30.01	51.57 ± 29.16

Values given as mean ± SD. Physical activity level (Low PA group: 0 ~ 70 minutes/week, middle PA group: 71 ~ 165 minutes/week, high PA group: 166 ~ 540 minutes/week). *Significant difference with low PA.

### Descriptive analysis of oncologists’ exercise recommendations

Forty-five percentages of oncologists initiated a discussion about their patients’ exercise during the last month. The average duration of discussion of exercise by oncologists during an office visit was five minutes. The three most recommended exercise types were aerobic exercise, flexibility exercises, and lifestyle modification. The two benefits oncologists most expected from exercise were improvements in mental health and improvements in ability to perform daily tasks. The three most prominent barriers oncologists encountered in recommending exercise to their patients were lack of time during office visits, unclear exercise guidelines for cancer patients, and concerns about the safety of their patients (Table 4).

**Table 4:** **Descriptive analysis of oncologists’ exercise recommendations**

Variable		Mean ± SD
What percentage of your patients have initiated a discussion with you about exercise during cancer treatment over the past month?		29.5 ± 22.4
What percentage of your patients have you recommended exercise to during cancer treatment over the past month?		45.0 ± 28.2
On average, if a patient initiates a discussion with you on exercise, how long do you spend discussing this topic? (min)		4.3 ± 2.9
Benefits of exercise for cancer survivors (Multiple response)		
	N	%
Improve the ability to perform daily tasks	128	26.7
Improve mental health	118	24.6
Attenuate physical decline from treatment	92	19.2
Reduce body weight	44	9.2
Reduce the risk of other diseases	37	7.7
Help patients cope	34	7.1
Reduce the risk of recurrence	23	4.8
No recommendation	2	0.4
Other	1	0.2
Total	479	100
What kind of exercise do you recommend (Multiple response)		
	N	%
Aerobic activity	157	57.1
Flexible activity	58	21.1
Lifestyle activity	48	17.5
Resistance activity	11	4.0
Total	275	100
Barriers to recommending exercise for cancer survivors (Multiple response)		
	N	%
Lack of time during office visit	40	24.0
Unclear recommendations	35	21.0
Concerns about the safety of exercise	34	20.4
Lack of patient interest	13	7.8
Concerns about the effectiveness of exercise	7	4.2
Lack of reimbursement for counseling on exercise	6	3.6
Enough recommendations	32	19.2
Total	479	100

Values given as mean ± SD for continuous variables and frequency (%) for categorical variables.

### The impact of perceived benefits of exercise and barriers to recommending exercise on actual exercise recommendations

**Figure 1:**
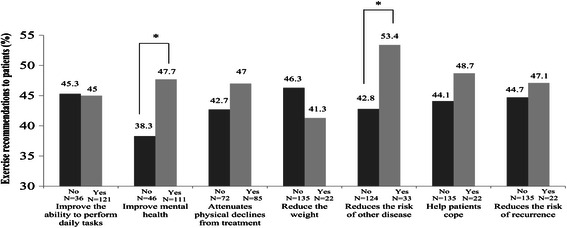
**Exercise recommendations across benefits of exercise for cancer survivors.** Figure legend: *p < .05 for difference within group. Oncologists who believe the benefit of exercise on each questionnaire item chose ‘yes’ while those who did not believe the benefit of exercise on each questionnaire item chose ‘no’. Y axis represent percentage of exercise recommendation to their patients.

**Figure 2:**
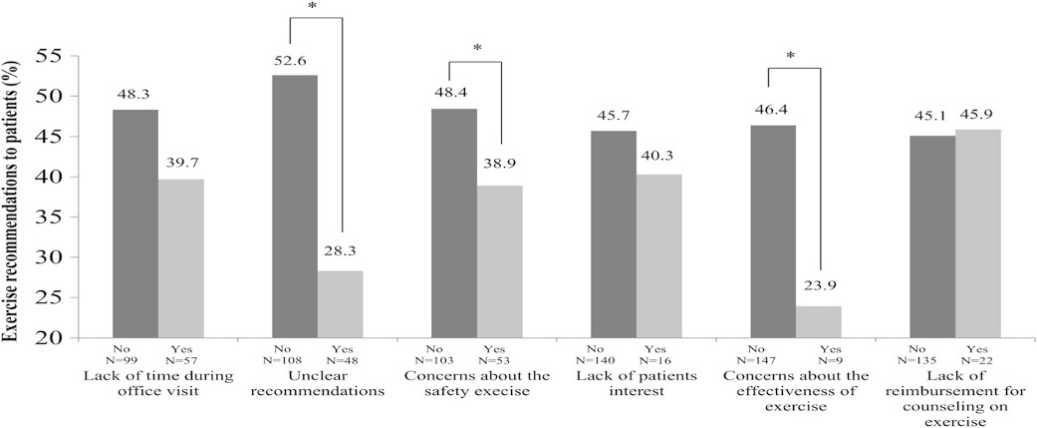
**Exercise recommendations across barriers to recommending exercise for cancer survivors.** Figure legend: *p < .05 for difference within group. Oncologists who believe the barriers of exercise on each questionnaire item chose ‘yes’ while those who did not believe the benefit of exercise on each questionnaire item chose ‘no’. Y axis represent percentage of exercise recommendation to their patients.

To understand the impact of oncologists’ perceived benefits of exercise on exercise recommendations, we analyzed the percentage of oncologists’ exercise recommendation based on their perceived benefits of exercise. Our analysis showed that oncologists who believe that participation in exercise will improve their patients’ mental health as well as reduce the risk of other diseases recommended exercise more (Figure 1). To understand the association between exercise recommendation barriers and exercise recommendations, we analyzed the percent of oncologists’ exercise recommendation based on items of oncologists’ exercise recommendation barriers. Our analysis showed that oncologists who have concerns regarding unclear exercise guidelines for cancer patients and the effectiveness of exercise for cancer patients recommended exercise to their patients significantly less (Figure 2).

## Discussion

Previous studies demonstrated that cancer patients tend to exercise more if they receive exercise recommendations from their oncologists [16,17,21,22]. In the present study, we have identified the characteristics of exercise recommendations among oncologists, the characteristics of exercise recommendations based on oncologists’ own physical activity levels, the perceived benefits of exercise among oncologists, and the barriers oncologists perceive to recommending exercise to their patients. Most oncologists believe that exercise during treatment is beneficial and important for cancer patients. However, fewer oncologists agreed that exercise during treatment is safe or easy. Furthermore, most oncologists thought very positively about recommending exercise to their patients during treatment. Only a small number of oncologists thought negatively about recommending exercise to their cancer patients. However, just 7.2% of oncologists agree that their cancer patients manage to exercise during cancer treatment. Therefore, our findings show, first, that most oncologists think that exercise during cancer treatment is beneficial and important but that they have concerns about the safety of exercise, and, second, oncologists thought that very few of their patients are actually exercising during cancer treatment in Korea.

Unlike the opinions of the oncologists in the current study that only 7.2% of cancer patients manage to exercise during cancer treatment, a previous study showed that 37% of colorectal cancer survivors and 28% of breast cancer survivors participate in regular physical activity during treatment [18,28]. Furthermore, 32% of breast cancer survivors actually participated in recommended levels of physical activity, defined as 150 min per week of moderate to vigorous intensity sports/recreational physical activity, after the completion of treatment [15]. Blanchard and colleagues examined the prevalence of physical activity in 9105 cancer survivors [29] and reported that 30–47% of survivors of cancer are meeting the physical activity recommendations. In Korean cancer patients, we have recently determined that 7.6% of Korean colorectal cancer patients participated in more than ACSM guidelines (150 min per week of moderate to vigorous physical activity) during cancer treatment, significantly lower patients than the 20.5% who participated physical activity more than ACSM guideline during before the cancer diagnosis [18]. However, the reason why the percentage of individuals meeting ACSM guidelines decreased during treatment is due to reduced vigorous intensity of physical activity, while the amount of mild intensity physical activity tended to increase during treatment (111.1 ± 203.9 min versus 146.8 ± 232.2 min) compared to before cancer diagnosis [18]. Although there is a possibility of over reporting of the level of physical activity among cancer survivors [30], the results of the current study may suggest that oncologists in Korea may underestimate the exercise ability of their cancer patients.

The attitudes of oncologists toward exercise and toward recommending exercise are very important since the exercise recommendations of oncologists are a strong predictor of cancer patients’ participation in exercise [31]. Previous studies indicated that cancer patients are highly motivated to receive advice on exercise and that they consider their oncologist an important source of this information [17,19]. Jones and colleagues [19] surveyed 311 survivors of prostate, breast, colorectal, or lung cancer, and a total of 84% of the participants indicated that they would prefer to receive exercise counseling during their cancer experience. Our study showed that over 70% of oncologists believed that exercise is important and beneficial to cancer patients. Our study further showed that 87% of oncologists believed that providing exercise recommendations to cancer patients is not hard, but only 40% of oncologists actually recommended exercise to their patients in the past one month. These results are similar to those of previous studies, which found that approximately 43% and 44% of oncologists regularly provided exercise recommendations and/or discussed exercise with their patients when appropriate [16,21].

To understand whether individual physical activity participation levels influence exercise recommendations and attitudes toward exercise recommendations, we also examined the oncologists’ own physical activity levels. Our analysis showed that only 11.9% of the surveyed oncologists met ACSM physical activity guidelines, a rate notably lower than that for oncologists of several other countries, such as the 52.5% of Canadian oncologists [21] or 57.3% of American oncologists [25] who met the guidelines. Our analysis also showed that the oncologists’ own physical activity participation was associated with their attitude toward exercise recommendation to their patients. Those who participated in more physical activity believe that their patients think that they should recommend exercise; they also think that recommending exercise is dependent on them and they tried to recommend exercise to their patients more.

In our study, we also surveyed the oncologists’ perceived benefits of exercise for cancer survivors. They believed that exercise participation would improve the ability of patients to perform daily tasks (26.7%), improve mental health (24.6%), attenuate physical decline from treatment (19.2%), reduce body weight (9.2%), and reduce the risk of other diseases (7.7%). It is interesting that only 4.8% of oncologists actually think that exercise may reduce cancer recurrence, while many reported studies have shown that exercise reduced the recurrence of various cancers [12,13].

A growing number of large observational studies have reported that physical activity reduces all-cause and cancer-specific mortality, which suggests that exercise may confer additional improvements in breast and colorectal cancer survival beyond surgery [32-34]. Holmes et al. [32] demonstrated that women with breast cancer who participated in more than 9 metabolic equivalent (MET) hours per week of physical activity have a 41%, 50%, and 43% reduction in the risk of total death, breast cancer death, and risk of recurrence, respectively, compared with those who participated in fewer than 3 MET hours per week of physical activity. Meyerhardt et al. [35] also demonstrated that more than 18 MET hours per week of physical activity after diagnosis is associated with a 45-61% reduction in the risk of colorectal cancer-specific death and a 57-63% reduction in the risk of total death. Although it is beyond the scope of our study, it may be interesting to ascertain whether knowledge of the impact of physical activity on cancer recurrence and mortality would influence the oncologists’ patterns of physical activity and exercise recommendations. In our study, we analyzed whether oncologists’ perceived benefits of exercise for cancer patients would be associated with their exercise recommendations. We observed that oncologists who believe that exercise will improve patients’ mental health as well as reduce the risk of other diseases recommended exercise to their patients significantly more than those who do not think exercise will have such benefits for their patients. Based on these results, we can speculate that knowledge of the impact of exercise on cancer patients may also influence the patterns and characteristics of exercise recommendations. The result of our study may suggest that the information on the validated benefit of exercise for cancer survivors should be easily available for oncologists to increase their exercise recommendation to patients more. Since cancer survivors’ exercise/physical activity behavior is easily influenced by oncologists’ exercise and physical activity recommendation, difference in exercise recommendation percentage of oncologists may actually have clinical relevance.

In assessing the factors which make it difficult to recommend exercise, lack of time during office visits, unclear recommendations, and concerns about the safety of exercise were the three most prevalent answers. Herbert et al. [36] reported that the most common barriers to providing physical activity counseling by primary care provider’ to patients in a clinical setting were lack of time, lack of knowledge, and lack of success in changing patient behavior. Not having sufficient time during office visits is a significant barrier to recommending exercise for many oncologists, since doctors spend, on average, less than 5 minutes during an office visit [37]. However, knowing that oncologists’ 30-second exercise recommendation increased patients’ physical activity level by 3.4 MET hours per week in breast cancer patients [17], it would be important to develop and provide an exercise recommendation tool to assist oncologists in providing effective exercise recommendations in a short time. Unclear recommendations and concerns about safety, meaning unclear guidelines for cancer patients, are also significant barriers for oncologists in recommending exercise to their patients. The ACSM Roundtable on Exercise Guidelines for Cancer Survivors [14] concluded that exercise training is safe during and after cancer treatments and results in improvements in physical functioning, quality of life, and cancer-related fatigue in several cancer survivor groups. In addition, other studies in cancer types including breast cancer, colorectal cancer, and prostate cancer have continually shown that exercise for cancer patients during and after treatment is safe [14]. In our analysis, we found that oncologists who were unclear about exercise recommendations and who had concerns about the safety of exercise recommended exercise to their patients significantly less. Therefore, it is important to provide information on the safety of exercise in cancer patients to oncologists.

Although the present study provides important information regarding oncologists’ attitudes toward exercise, there are several limitations that need to be considered when interpreting the results of this study. A selection bias is likely to exist because of the transparent purpose of the study, and the relatively low response rate to our survey may limit the generalizability of our findings. Furthermore, a possible response bias is another limitation; oncologists who were more interested in physical activity were probably more likely to respond. Another important limitation is the use of a retrospective observational design that provides the weakest evidence in terms of causality and is susceptible to memory biases. As such, it is possible that oncologists may have recalled numerous discussions with patients interested in exercise and not those with all patients, which therefore would overestimate the actual recommendation rates. Future research is required using prospective designs with objective measures of a patient–oncologist discussion of exercise (e.g., audiotaped consultation review). Lastly, since the current study is a cross-sectional study, it is not possible to identify the cause and effect relationship between variables.

## Conclusion

Our findings demonstrate that oncologists have favorable attitudes toward and interest in exercise, although significant barriers may prevent oncologists from providing exercise advice to their patients. As the importance of exercise in oncology settings continues to grow, it appears inevitable that oncologists will become an important source of exercise information. Therefore, it is important to develop exercise guidelines with an information package which oncologists can easily use to provide exercise recommendations to their patients. As such, further research is required evaluating the effectiveness and efficacy of interventions and strategies designed to improve oncologists’ confidence and knowledge to provide effective and safe exercise recommendations to their patients during oncology consultations.

## Abbreviations

SD: Standard deviation
ACSM: American College of Sports Medicine
GLTEQ: Godin leisure -time exercise questionnaire
MET: Metabolic equivalent
